# Hepatitis C Virus Induces E6AP-Dependent Degradation of the Retinoblastoma Protein

**DOI:** 10.1371/journal.ppat.0030139

**Published:** 2007-09-28

**Authors:** Tsubasa Munakata, Yuqiong Liang, Seungtaek Kim, David R McGivern, Jon Huibregtse, Akio Nomoto, Stanley M Lemon

**Affiliations:** 1 Center for Hepatitis Research, University of Texas Medical Branch, Galveston, Texas, United States of America; 2 Department of Microbiology, Graduate School of Medicine, University of Tokyo, Tokyo, Japan; 3 Department of Microbiology and Immunology, University of Texas Medical Branch, Galveston, Texas, United States of America; 4 Department of Molecular Genetics and Microbiology, Institute for Cellular and Molecular Biology, University of Texas Austin, Austin, Texas, United States of America; 5 Sealy Center for Cancer Cell Biology, University of Texas Medical Branch, Galveston, Texas, United States of America; The Salk Institute for Biological Studies, United States of America

## Abstract

Hepatitis C virus (HCV) is a positive-strand RNA virus that frequently causes persistent infections and is uniquely associated with the development of hepatocellular carcinoma. While the mechanism(s) by which the virus promotes cancer are poorly defined, previous studies indicate that the HCV RNA-dependent RNA polymerase, nonstructural protein 5B (NS5B), forms a complex with the retinoblastoma tumor suppressor protein (pRb), targeting it for degradation, activating E2F-responsive promoters, and stimulating cellular proliferation. Here, we describe the mechanism underlying pRb regulation by HCV and its relevance to HCV infection. We show that the abundance of pRb is strongly downregulated, and its normal nuclear localization altered to include a major cytoplasmic component, following infection of cultured hepatoma cells with either genotype 1a or 2a HCV. We further demonstrate that this is due to NS5B-dependent ubiquitination of pRb and its subsequent degradation via the proteasome. The NS5B-dependent ubiquitination of pRb requires the ubiquitin ligase activity of E6-associated protein (E6AP), as pRb abundance was restored by siRNA knockdown of E6AP or overexpression of a dominant-negative E6AP mutant in cells containing HCV RNA replicons. E6AP also forms a complex with pRb in an NS5B-dependent manner. These findings suggest a novel mechanism for the regulation of pRb in which the HCV NS5B protein traps pRb in the cytoplasm, and subsequently recruits E6AP to this complex in a process that leads to the ubiquitination of pRb. The disruption of pRb/E2F regulatory pathways in cells infected with HCV is likely to promote hepatocellular proliferation and chromosomal instability, factors important for the development of liver cancer.

## Author Summary

Persons infected with hepatitis C virus (HCV) are at increased risk for liver cancer. This is remarkable because HCV is an RNA virus with replication confined to the cytoplasm and no potential for integration of its genome into host cell DNA. While it is likely that chronic inflammation contributes to liver cancer, prior studies with HCV transgenic mice indicate that the viral proteins are intrinsically carcinogenic. In this study, we have examined the interaction of one of these, the RNA-dependent RNA polymerase nonstructural protein 5B, with an important cellular tumor suppressor protein, the retinoblastoma protein (pRb). pRb is a master regulator of the cell cycle, and altered expression of some of the many genes it regulates may lead to cancer. We show that the abundance of pRb is strongly downregulated in cells infected with HCV, and that nonstructural protein 5B targets pRb for destruction via the cell's normal protein degradation machinery. The E6-associated protein appears to play a role in this process, which is interesting as it also mediates the degradation of another tumor suppressor, p53, by papillomaviruses. The loss of pRb function in HCV-infected cells likely promotes hepatocellular proliferation as well chromosomal instability, factors important for the development of liver cancer.

## Introduction

Among viruses that infect the human liver, hepatitis C virus (HCV) is a leading cause of morbidity and mortality worldwide [1]. Chronic infection with HCV is a major risk factor for the development of cirrhosis as well as hepatocellular carcinoma (HCC) [2,3]. The incidence of this cancer has increased dramatically in recent years in Japan and the United States, reflecting prior increases in the prevalence of HCV infection, and in Japan HCV has replaced hepatitis B virus as the leading infectious cause of liver cancer. The strong association between HCC and HCV infection is particularly notable in that HCV is a positive-strand RNA virus, classified within the genus *Hepacivirus* of the family *Flaviviridae* [4]. Its 9.6-kb genome replicates in association with membranes within the cytoplasm of infected cells, and encodes a single polyprotein that is processed by both cellular and viral proteases into ten individual structural and nonstructural viral proteins.

Although inflammation associated with chronic hepatitis C is likely to contribute to the development of HCC, there is strong evidence that one or more of the proteins expressed by the virus contribute directly to carcinogenesis. The HCV core protein, a component of the putative viral nucleocapsid, has been shown to modulate the hepatocyte cell cycle [5,6]. Other studies suggest that expression of the nonstructural (NS) proteins, NS3 (a serine proteinase/helicase), NS5A (a replicase-associated phosphoprotein of uncertain function), or NS5B (the viral RNA-dependent RNA polymerase) may also affect control of cellular proliferation [7–10]. Moreover, transgenic mice expressing a high abundance of the core protein develop steatosis and HCC [11]. Liver cancer also developed in transgenic mice expressing a much lower abundance of the entire viral polyprotein, but not in a companion transgenic lineage expressing a higher abundance of the structural proteins (core, E1, E2, and p7) only [12]. None of these transgenic mouse lineages had demonstrable hepatic inflammation in advance of the development of HCC. Together, these data suggest a direct role for both structural and nonstructural HCV proteins in oncogenesis.

At least four different pathways that regulate either cell proliferation or cell death, the retinoblastoma (pRb)/E2F, p53, transforming growth factor-β (TGF-β), and β-catenin pathways, are commonly altered in HCCs [2]. Among them, pRb plays a major role in controlling the G_1_- to S-phase transition and mitotic checkpoints through a repressive effect on E2F transcription factors [13]. pRb functions as a tumor suppressor, and the gene which encodes it (*RB*) is frequently mutated in various types of tumors, including retinoblastomas, small-cell lung carcinomas, and osteosarcomas [14]. In previously published studies, we demonstrated that pRb protein abundance is negatively regulated in cells supporting the replication of subgenomic and genome-length HCV RNA replicons [8]. Similar to the DNA virus oncoproteins E1A of adenovirus and E7 of human papillomavirus (HPV), we found that the RNA-dependent RNA polymerase of HCV, NS5B, forms a complex with pRb in these cells, targeting pRb for degradation and resulting in a reduction in its abundance. This leads to the activation of E2F-responsive promoters in cells containing HCV RNA replicons, and promotes progression of the cell cycle from G_1_- to S-phase in cells expressing NS5B [8]. While potentially important with respect to the development of HCC in people with chronic hepatitis C, the molecular mechanisms underlying these observations have not been characterized.

Here, we show that pRb is downregulated not only in cells bearing HCV RNA replicons, but also in human hepatoma cells infected in vitro with different genotypes of HCV. We demonstrate that the downregulation of pRb occurs via the ubiquitin-proteasome system, and that it is dependent upon the activity of a known E3 ubiquitin ligase, E6-associated protein (E6AP). E6AP forms a complex with NS5B and pRb, and pRb is ubiquitinated in an NS5B-dependent manner. Our findings reveal a novel mechanism that regulates pRb abundance in HCV-infected hepatocytes, and offer an enhanced understanding of the events leading to the development of HCC in chronically infected patients.

## Results

### HCV Infection Downregulates pRb and Phospho-pRb Expression In Vitro

**Figure 1:**
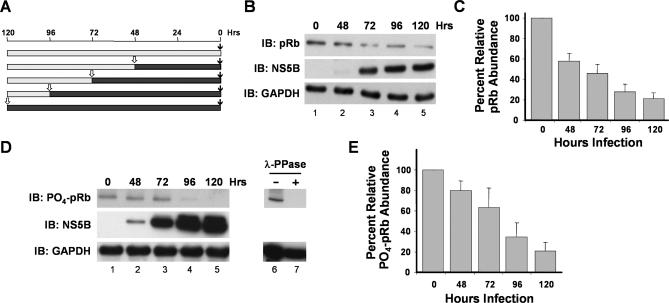
Infection of Cultured Human Hepatoma (Huh-7.5) Cells Leads to Downregulation of pRb Abundance (A) Schema for experiment shown in (B–E). Huh-7.5 cells were seeded into culture vessels and infected with JFH1 virus (MOI ∼1) at intervals, and subsequently lysed simultaneously for immunoblot analysis. Solid bars show infected; shaded bars, uninfected. (B) Immunoblots for total pRb and NS5B in cells infected with JFH1 virus according to the protocol outlined in (A). GAPDH was used as a loading control. (C) Mean ± range of values for abundance of pRb in cells infected with JFH1 virus in replicate experiments, relative to abundance in uninfected cells. (D) Immunoblots for phospho-pRb (residues 807/811) and NS5B in cells infected with JFH1 virus. GAPDH was used as a loading control. Lanes 6–7 demonstrate the specificity of the phospho-pRb: lysate from uninfected Huh-7.5 cells was subjected to immunoblotting before (lane 6) and after (lane 7) digestion with lambda protein phosphatase (λ-PPase). (E) Mean ± range of values for abundance of phospho-pRb (residues 807/811) in cells infected with JFH1 virus in replicate experiments, relative to abundance in uninfected cells.

We demonstrated previously that the abundance of pRb is downregulated post-transcriptionally in cells supporting replication of both subgenomic and genome-length HCV RNA replicons derived from a genotype 1b strain of HCV, HCV-N [8]. However, these earlier studies did not determine whether pRb is also downregulated during the course of HCV infection, because genome-length HCV-N RNA is not capable of producing virus that is infectious in cultured cells [15]. To address this question, we used a genotype 2a virus strain, JFH1, that is capable of undergoing the complete viral life cycle in Huh-7 cells in vitro [16–18]. pRb abundance was reduced significantly within 48–72 h of infection with JFH1 virus (Figure 1A and 1B), with quantitative analysis of immunoblots indicating that the pRb abundance 120 h after infection was approximately 20%–30% that of uninfected cells (Figures 1C and S1). The activity of pRb is normally regulated through its phosphorylation by cyclin-dependent kinases, and we also observed an equivalent reduction in the abundance of phospho-pRb in JFH1-infected cells using an antibody specific for pRb phosphorylated at residues 807/811 (Figure 1D and 1E).

**Figure 2:**
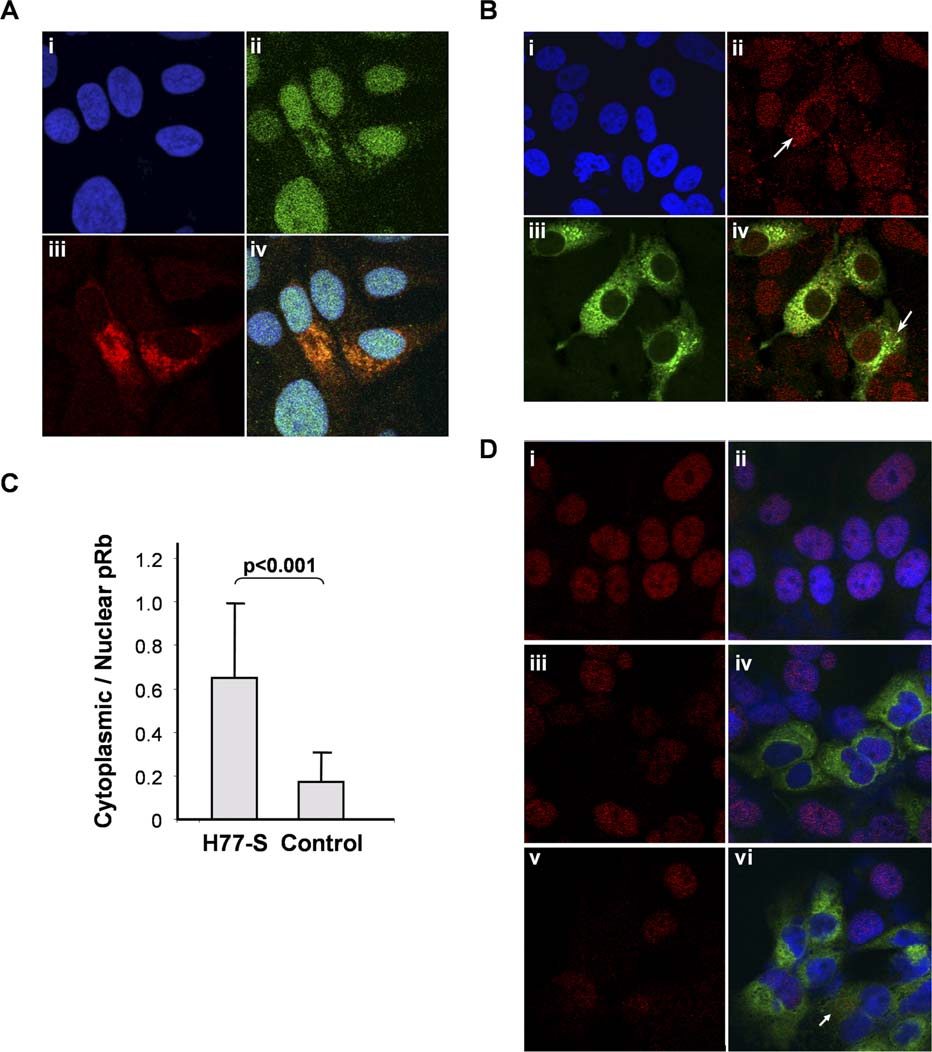
Confocal Microscopic Images Demonstrated Alterations in pRb and Phospho-pRb Expression Associated with HCV Infection of Huh-7.5 Human Hepatoma Cells (A) pRb accumulates in the cytoplasm of cells 48 h after infection with genotype 2a JFH1 virus. Cells were labeled with polyclonal antibody to the HCV NS5A protein (Alexa-594; red) and monoclonal anti-pRb (fluorescein; green), and counterstained with DAPI (blue) to visualize nuclei. Two cells near the center of the field are infected with virus and show cytoplasmic displacement of pRb. Frames: i, DAPI only; ii, pRb; iii, NS5A; iv, merge. (B) Confocal microscopic images of Huh-7.5 cells 84 h after infection with the genotype 1a H77S virus [20] at low MOI. Labeling of HCV antigens with polyclonal human antibody (fluorescein; green) revealed both infected and uninfected cells. pRb was labeled with monoclonal antibody (Alexa-568; red), and in infected cells has been either partially (frame iv, arrow) or completely (frame ii, arrow) redirected to the cytoplasm. Nuclei were visualized by DAPI staining. Frames: i, DAPI only; ii, pRb; iii, HCV; iv, merge. (C) The cytoplasmic and nuclear abundance of pRb was determined quantitatively in confocal microscopic images of H77S virus–infected Huh-7.5 cells using MetaMorph software (Molecular Devices, http://www.moleculardevices.com/). The average integrated intensities of the pRb fluorescence signal were determined for both the cytoplasm and nucleus (delineated by DAPI staining) in individual infected and immediately adjacent uninfected cells. These data were used to calculate a cytoplasmic–nuclear pRb ratio for each individual cell. Infected cells (identified by labeling of HCV antigens as in [A]) and uninfected cells were analyzed separately, revealing a striking difference in the ratio of cytoplasmic–nuclear pRb staining intensity in infected (0.65) versus uninfected (0.17) cells (*p* < 0.001 by Student *t* test). (D) Confocal microscopy images of phospho-pRb expression in uninfected Huh-7.5 cells (frames i and ii), and 96 h following infection with the genotype 2a JFH1 virus (frames iii–vi). Frames i, iii, and v show labeling with monoclonal antibody to phospho-pRb 807/811 (red) only, whereas frames ii, iv, and vi show merged images of phospho-pRb (red), HCV (human polyclonal antibody; green), and DAPI labeling. The red channel gain was increased in frames iii–vi in order to normalize the intensity of phospho-pRb labeling within the nuclei of noninfected cells with that in frames i and ii. Nuclear phospho-pRb expression was ablated by JFH1 infection, and only partially restored after 20 h of treatment with 50 nM epoxomicin (frames v and vi). The arrow in frame vi indicates an accumulation of phospho-pRb within the cytoplasm of an infected cell following epoxomicin treatment.

Coincident with the reduction in total pRb abundance, confocal microscopy demonstrated a striking cytoplasmic relocalization of pRb in JFH1-infected cells (Figure 2A). Our earlier studies demonstrated that expression of the NS5B RNA polymerase is responsible for the downregulation of pRb in replicon-bearing cells, and that NS5B interacts directly with pRb through a Leu-X-Cys-X-Glu homology domain (LH^314–318^) overlapping the polymerase active site [8]. Since we lack antibody capable of labeling the genotype 2a NS5B protein within infected cells, we labeled the viral replicase complex in these cells with a broadly reactive polyclonal antibody to NS5A. NS5A is known to colocalize with HCV RNA and other HCV nonstructural proteins within the cytoplasmic “membranous webs” that are thought to be sites of viral RNA synthesis [19]. These studies revealed numerous JFH1-infected cells with an abnormal cytoplasmic accumulation of pRb, and a clear colocalization with the HCV replicase complex as labeled with anti-NS5A (Figure 2A, frame iv). Although the sensitivity of confocal microscopy for detection of pRb renders it difficult to deduce quantitative differences in nuclear pRb abundance in infected versus noninfected cells, we noted similar cytoplasmic relocalization of pRb in cells infected with a cell culture–infectious genotype 1a virus (H77S) developed recently in our laboratory [20] (Figure 2B). Some H77S-infected cells demonstrated nearly complete relocalization of pRb to the cytoplasm (Figure 2B, frame ii arrow), while in most infected cells, there was partial cytoplasmic relocalization (Figure 2B, frame iv arrow). A quantitative analysis of multiple cells indicated that there was a significant increase in the proportion of total pRb present in the cytoplasm of infected versus noninfected cells (Figure 2C; *p* < 0.001). Confocal microscopy also confirmed a striking reduction in nuclear phospho-pRb in JFH1-infected cells (Figure 2D; compare frames i and iii). Phospho-pRb was found to accumulate within the cytoplasm following treatment with epoxomicin (Figure 2D, frame vi). These results are consistent with an interaction occurring between NS5B and pRb within the cytoplasm. While pRb is normally confined to the nucleus, it does undergo nuclear-cytoplasmic shuttling involving phosphorylation-dependent nuclear export mediated by exportin1 [21]. It is likely that NS5B interacts with and traps pRb in the cytoplasm prior to its initial transport to the nucleus, or perhaps after nuclear export.

### HCV Promotes Proteasome-Dependent Degradation of pRb

**Figure 3:**
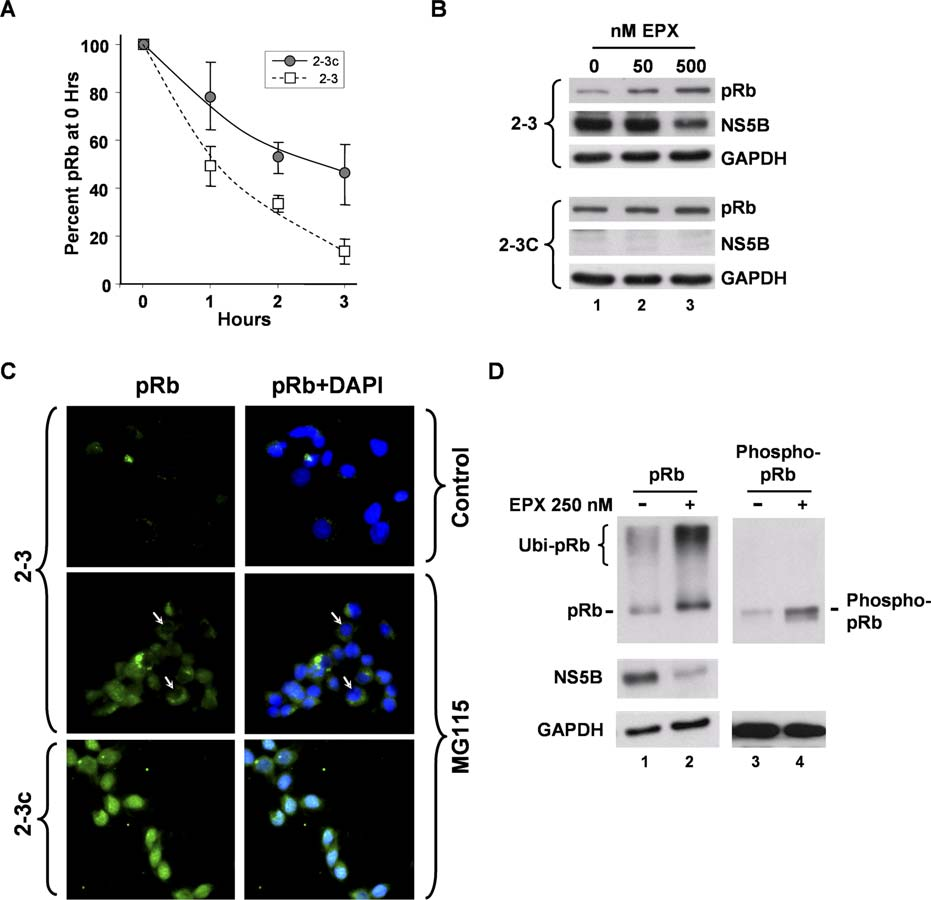
HCV Promotes Proteasome-Mediated Degradation of pRb (A) Pulse-chase labeling of pRb in 2–3 replicon (squares) and cured 2–3c (circles) cells. Cells were pulse labeled with [^35^S]-methionine/cysteine for 30 min, then chased with DMEM supplemented with cysteine and methionine and lysed at the times indicated. pRb was immunoprecipitated from lysates and separated by SDS-PAGE. [^35^S] was quantified by PhosphoImager analysis. Results represent the mean ± range % [^35^S] label recovered from each cell type at 0 h. (B) Inhibition of the proteasome with epoxomicin (EPX) partially restores pRb abundance in NNeo-C5B/2–3 cells containing an autonomously replicating, genome-length HCV RNA replicon. 2–3 cells and their interferon-cured, HCV-negative progeny, 2–3c cells [58], were treated with 0, 50, or 500 nM EPX for 20 h, followed by lysis and immunoblot analysis of pRb and NS5B. GAPDH was used as a loading control. (C) Inhibition of the proteasome with MG115 increases cytoplasmic pRb abundance in 2–3 replicon cells. 2–3 (top and middle frames) and 2–3c (lower frames) cells were treated with 0 μM (top frames) or 20 μM (middle and lower frames) MG115 for 8 h prior to fixation, labeling with anti-pRb and DAPI, and examination with a fluorescence microscope. Frames on the left show pRb labeling (fluorescein; green), while frames on the right show merged pRb and nuclear (DAPI; blue) labeling. Arrows denote MG115-treated 2–3 cells in which pRb expression is entirely cytoplasmic, with no apparent nuclear staining. (D) EPX treatment restores (lanes 1–2) pRb and (lanes 3–4) phospho-pRb abundance, and increases the abundance of high-molecular-mass pRb-immunoreactive protein (probable polyubiquitinated pRb) in HCV-infected cells. Cells were infected for 96 h with JFH1 virus and treated with 250 nM EPX for 20 h prior to lysis and immunoblot analysis.

Although our previous studies demonstrated that NS5B downregulates the abundance of pRb post-transcriptionally [8], the mechanism by which this occurs is not clear. We confirmed our earlier observations that the stability of pRb is reduced in cells supporting HCV RNA replication by carrying out additional pulse-chase labeling experiments in Huh-7 2–3 cells which contain a genome-length RNA replicon [22]. These results indicated a significantly shortened half-life for pRb in the replicon cells, compared with clonally related cells (2–3c cells) from which the HCV RNA had been eliminated by prior interferon treatment (Figure 3A).

Since the abundance of pRb is regulated through proteasome-dependent pathways in the absence of HCV protein expression [23,24], we considered it likely that HCV may also regulate pRb in a proteasome-dependent fashion. To test this hypothesis, we determined whether proteasome inhibitors [25,26] could restore the abundance of pRb in 2–3 replicon cells. As shown in Figure 3B, epoxomicin, a potent and selective synthetic inhibitor of multiple protease activities of the proteasome, caused a marked increase in pRb abundance, nearly to normal levels, in 2–3 cells (Figure 3B, top panels). In contrast, epoxomicin treatment caused a slight increase in pRb abundance in the cured, HCV-negative 2–3c cell line (Figure 3B, lower panels), consistent with the normal regulation of pRb abundance by proteasomal degradation [23,24]. We also observed similar, cell-type–specific restoration of pRb abundance in 2–3 cells after treatment with lactacystin, an irreversible inhibitor of the 20S proteasome, or MG115, a reversible inhibitor of 20S and 26S proteasomes (Figure S2). Immunofluorescence analysis confirmed the rescue of pRb expression following MG115 treatment of 2–3 replicon cells, but revealed that pRb was localized primarily within the cytoplasm, and not the nucleus, in most 2–3 cells following treatment with the proteasome inhibitor (Figure 3C, arrows). Importantly, pRb retained its normal nuclear localization in MG115-treated 2–3c cells that lack HCV protein expression (Figure 3C, bottom panels). The retention of pRb in the cytoplasm of MG115-treated replicon cells (Figure 3C) is consistent with the interaction of NS5B discussed above. This interaction appears to trap the tumor suppressor protein within the cytoplasm and prevent its translocation to the nucleus in advance of its degradation by the proteasome.

To determine whether inhibition of the proteasome would similarly rescue pRb expression in cells infected with HCV, we treated JFH1-infected cells with epoxomicin. As shown in Figure 3D, this resulted in a marked increase in the abundance of pRb (compare lanes 1 versus 2), as well as an increase in high-molecular-mass pRb-immunoreactive protein. While the identity of this high-mass pRb-immunoreactive protein is uncertain, it is likely to represent ubiquitinated pRb (see below). The abundance of phospho-pRb was also increased following epoxomicin treatment of JFH1-infected cells, although there we observed no discernable high-molecular-mass phospho-pRb species (Figure 3D, lanes 3 versus 4). Considered collectively, these results indicate that HCV regulates the abundance of pRb by promoting its proteasome-dependent degradation. Although overexpression studies have suggested that the NS5B polymerase itself may be regulated by polyubiquitination and proteasome-mediated degradation [27], epoxomicin treatment did not enhance, but rather reduced, the abundance of NS5B in both HCV-infected and replicon cells (Figures 3B and 3D).

### HCV-Dependent Polyubiquitination of pRb

**Figure 4:**
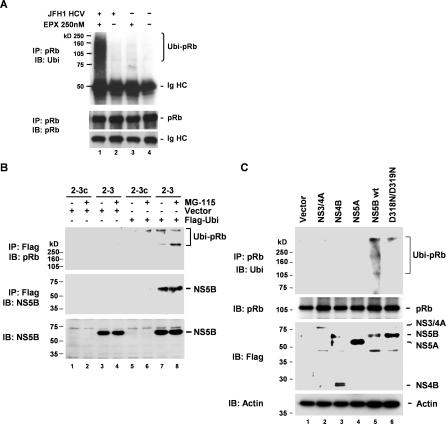
HCV Infection and RNA Replication Induces Polyubiquitination of pRb (A) Polyubiquitination of pRb in HCV-infected hepatoma cells. Huh-7.5 cells were infected with JFH1 virus (+) or mock-infected (−) for 96 h, and treated (+) or not treated (−) with EPX (250 nM) for 20 h prior to lysis, precipitation with anti-pRb, and immunoblotting with either anti-ubiquitin (Ubi; top panel) or anti-pRb antibody (bottom panels). (B) Polyubiquitination of pRb in HCV replicon cells. 2–3 and 2–3c cells were transfected with a Flag-ubiquitin expression vector and cultured in the presence or absence of MG115 prior to preparation of lysates. Lysates were immunoprecipitated with anti-Flag antibody, followed by immunoblotting with anti-pRb (top panel) or anti-NS5B (middle panel). The lower panel shows direct NS5B immunoblotting of the cell lysates. Ubiquitination of endogenous pRb is evident in the replicon cells even in the absence of MG115 treatment. NS5B is associated with the ubiquitinated Rb. (C) Ectopic expression of wild-type (wt) NS5B induces polyubiquitination of pRb in normal Huh-7 cells. Cells were transfected with vectors expressing Flag-tagged NS3-4A, NS4B, NS5A, NS5B, and a mutant NS5B, D318N/D319N, then treated with MG115. Cell extracts were immunoprecipitated with anti-pRb, followed by immunoblotting with anti-ubiquitin (top panel). The bottom panels show immunoblots of the cell extracts carried out with antibodies to pRb, Flag (expression control), and actin (loading control).

Although pRb abundance is normally regulated through proteasome-dependent pathways, such regulation does not necessarily require the ubiquitination of pRb [23,24]. The increase we observed in the abundance of high-molecular-mass pRb-immunoreactive protein in lysates of JFH1-infected hepatoma cells prepared following treatment with epoxomicin (Figure 3D, lane 2) suggests that HCV infection might promote the polyubiquitination of pRb. To assess this possibility, we immunoprecipitated pRb from lysates of Huh-7.5 cells that were infected with the JFH1 virus, then analyzed the precipitates in immunoblots using antibody to ubiquitin. We similarly studied infected cells that had been treated with epoxomicin for 20 h prior to lysis. These results revealed that HCV infection induces polyubiquitination of pRb (Figure 4A, lane 1). A significant abundance of polyubiquitinated pRb was not detected in lysates from mock-infected cells, even following treatment with epoxomicin (Figure 4A, compare lanes 3 and 1).

We also demonstrated HCV-dependent polyubiquitination of pRb in the 2–3 replicon cells by transfecting the cells with a vector expressing Flag-tagged ubiquitin, followed by immunoprecipitation (IP) of lysates with anti-Flag antibody and immunoblotting with anti-pRb (Figure 4B). While a small amount of polyubiquitinated pRb was detected in lysates of the cured 2–3c cells following treatment with MG115 (Figure 4B, lane 6), this was readily detected in lysates of untreated 2–3 replicon cells, and increased by MG115 treatment (Figure 4B, lanes 7–8). We obtained similar results in cells treated with lactacystin (unpublished data). Importantly, the anti-Flag precipitates from the 2–3 cells also contained abundant NS5B, indicating that NS5B was associated with the ubiquitinated pRb in these cells (Figure 4B, lanes 7–8). We observed no high-molecular-mass forms of NS5B, suggesting that there is no appreciable ubiquitination of NS5B under these conditions. These results confirm that HCV infection and/or RNA replication induce polyubiquitination of pRb as a prelude to its degradation by the proteasome.

Since our prior studies revealed that NS5B binds to and induces the degradation of pRb [8], we asked whether ectopic expression of NS5B would also induce pRb ubiquitination. We transfected normal Huh-7 cells with vectors expressing Flag-tagged HCV nonstructural proteins (NS3-4A, NS4B, NS5A, and NS5B), and immunoprecipitated cell extracts with anti-pRb, followed by immunoblotting with anti-ubiquitin antibodies. Overexpression of Flag-NS5B, but not other HCV nonstructural proteins, reproducibly resulted in polyubiquitination of pRb (Figure 4C). In contrast, the ectopic expression of an NS5B mutant (D318N/D319N) containing substitutions within the LH^314–318^ domain that mediates the interaction of NS5B with pRb [8] resulted in only minimal ubiquitination of pRb (Figure 4C; compare lanes 5 and 6). These data indicate that the interaction of NS5B with pRb leads to targeted destruction of pRb via the ubiquitin-proteasome pathway, representing a striking parallel to the mechanism by which the HPV E6 protein mediates destruction of p53 [28].

### HCV-Induced Degradation of pRb Is Dependent upon E6AP

A PROSITE (http://ca.expasy.org/prosite/) search indicated that the NS5B protein does not contain a RING finger, or a HECT or ubiquitin interaction motif, making it unlikely that NS5B itself possesses ubiquitin ligase activity [29]. Thus, NS5B is more likely to induce the ubiquitination of pRb by mediating its interaction with a cellular ubiquitin ligase. In searching for this protein, we focused on two recognized cellular E3 ubiquitin ligases: the human homolog of the murine “double minute 2” protein (MDM2), which is involved in ubiquitination pathways that normally regulate the abundance of pRb and p53 [23,24,30], and E6-associated protein (E6AP) which is recruited by the HPV E6 protein to mediate the ubiquitination of p53 [28]. Importantly, E6AP is also known to form a complex with ubiquilin-1 (hPLIC-1), a ubiquitin-like protein that has been shown to interact with NS5B [27,31]. Also, recent studies indicate that E6AP mediates the ubiquitination and degradation of the HCV core protein [32].

**Figure 5:**
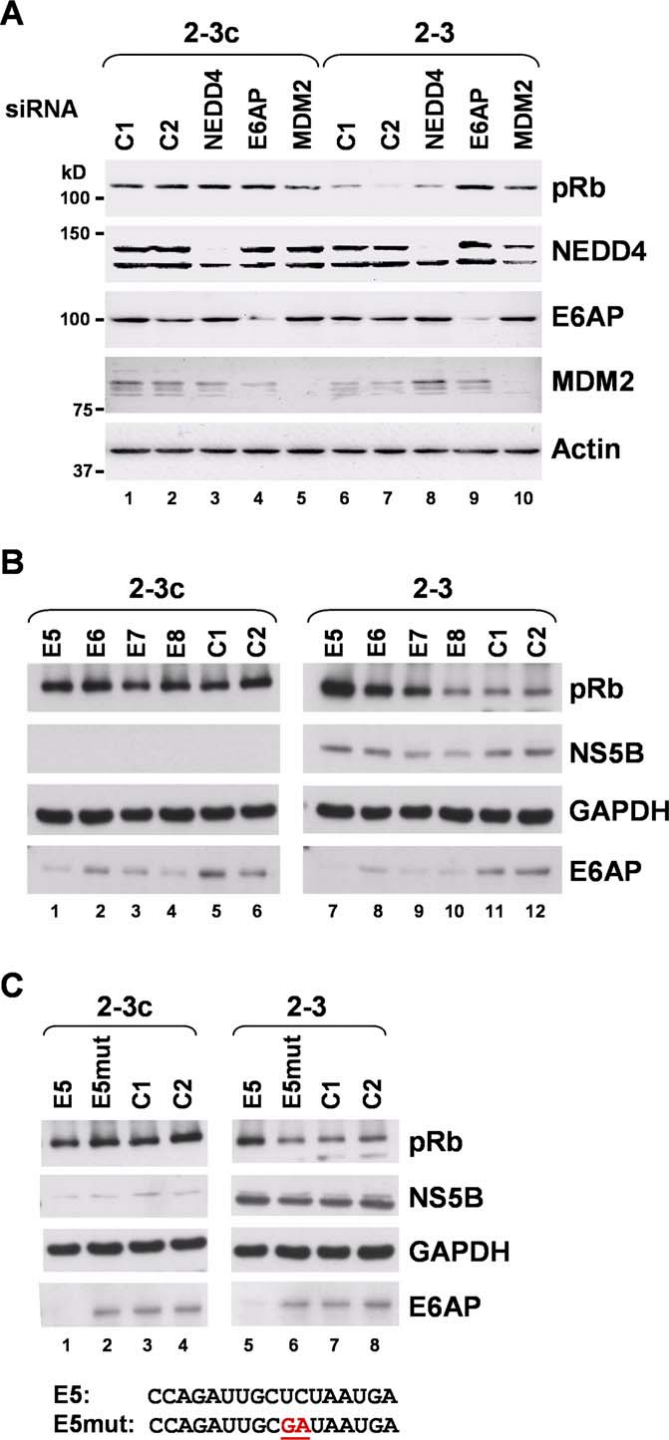
siRNA Knockdown of E6AP Restores pRb Abundance in HCV Replicon Cells (A) siRNA pools specific for the E3 ubiquitin ligases E6AP, MDM2, and NEDD4, and control siRNAs C1 and C2 were transfected into cured 2–3c cells (lanes 1–5) and 2–3 replicon cells (lanes 6–10) using a liposome-mediated procedure. Cell extracts were prepared 72 h later, separated by SDS-PAGE, and immunoblotted using antibodies to pRb and each of the target E3 ligases. While the each specific siRNA pool effectively reduced the abundance of its E3 ligase target, only the E6AP pool caused a reproducible restoration of pRb abundance in the 2–3 cells. (B) Effect of E6AP knockdown on pRb abundance using each of the four different E6AP-specific siRNAs (E5, E6, E7, and E8) comprising the siRNA pool tested in (A). C1 and C2 are control siRNAs. Lysates were prepared 120 h following transfection. (C) Transfection of a mutated E6AP siRNA (E5mut) fails to induce restoration of pRb abundance in 2–3 replicon cells. Nucleotide sequences of the E5 and E5mut siRNAs are shown at the bottom of the panel, with the two base substitutions underlined.

To assess the role of these two ubiquitin ligases in NS5B-mediated degradation of pRb, we used siRNA interference to examine the impact of MDM2 and E6AP knockdown on pRb abundance in the 2–3 replicon cells. Using pools consisting of four different specific siRNAs, we were able to achieve effective reductions of each targeted protein (Figure 5A). MDM2 knockdown resulted in a modest but variable increase in pRb abundance (Figure 5A; compare lane 8 with lanes 1 and 2), while knockdown of E6AP reproducibly restored pRb abundance to a level approaching that of the control 2–3c cells (Figure 5A; compare lane 9 with lanes 1 and 2). Two different control siRNAs failed to increase pRb abundance in the replicon cells (lanes 6 and 7). This was also the case with an siRNA pool specific for an E3 ligase sharing the C-terminal HECT domain of E6AP, neural precursor cell–expressed developmentally downregulated protein 4 (NEDD4) (lane 8) [33], indicating that the effect of E6AP knockdown was specific to that protein. None of the siRNAs tested significantly enhanced the abundance of pRb in the control, HCV-negative cells (Figure 5A, lanes 1–5).

To further assess the specificity of the E6AP knockdown, we transfected 2–3 and 2–3c cells with each of the four individual siRNA molecules present in the E6AP pool tested in Figure 5A. These results demonstrated robust enhancement of pRb abundance in cells transfected with three of the E6AP-specific siRNAs (E5, E6, and E7), while none of the siRNAs influenced pRb abundance in the control 2–3c cells (Figure 5B). Transfection of siRNA E8 reduced the abundance of E6AP, but had no apparent effect on pRb abundance (Figure 5B, lane 10). It is possible that this might reflect the involvement of an E6AP splicing variant, as the E5, E6, and E7 siRNAs all target exon 9 of the *E6AP* gene, while E8 targets exon 10 [34]. Alternatively, we observed kinetic differences in the rates of pRb restoration with these siRNAs, most likely reflecting different efficiencies of E6AP knockdown (unpublished data). Transfection of siRNAs E5 and E6 resulted in increased pRb abundance within 48 h, while this was not observed with siRNA E7 until 96 h or more after transfection. As a final proof of specificity, we mutated one of the E6AP-specific siRNAs (E5), altering the base at two consecutive positions (E5mut; Figure 5C, bottom). Compared with the wild-type E5 siRNA, transfection of E5mut resulted in neither knockdown of E6AP or restoration of pRb abundance (Figure 5C; compare lanes 5 and 6). Consistent with these results, overexpression of both E6AP and NS5B in Huh-7 cells enhanced the downregulation of pRb observed previously with ectopic expression of NS5B alone [8] (unpublished data).

### NS5B and pRb Form a Complex with E6AP

A hallmark of E6AP and other HECT domain ligases is their ability to form a physical complex with the molecule undergoing ubiquitination. Since the results described above suggest that E6AP may play a role in the NS5B-induced ubiquitination of pRb, we sought evidence for an interaction between E6AP, pRb, and NS5B.

**Figure 6:**
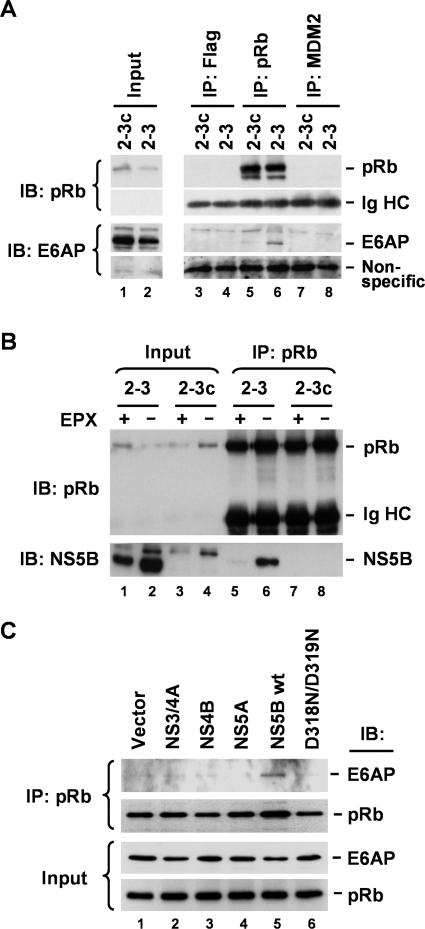
NS5B-Dependent Interaction between E6AP and pRb (A) Endogenous pRb interacts with E6AP in 2–3 replicon cells. Lysates from 2–3 and 2–3c cells were immunoprecipitated with anti-Flag (control), anti-pRb, or anti-MDM2 monoclonal antibody, followed by immunoblotting with anti-pRb and anti-E6AP. All monoclonal antibodies were derived from the same isotype, IgG_1_. Ig heavy chain (HC) and nonspecific bands served as loading controls for the pRb and E6AP immunoblots, respectively. (B) Endogenous pRb interacts with NS5B in 2–3 replicon cells in an EPX-sensitive fashion. 2–3 and 2–3c cells were cultured in the presence or absence of EPX prior to preparation of lysates. Lysates were immunoprecipitated with monoclonal anti-pRb antibody, followed by immunoblotting with anti-pRb and anti-NS5B antibodies. Note that EPX treatment reduces the apparent interaction between pRb and NS5B. (C) Ectopic expression of wild-type NS5B induces interaction between E6AP and pRb in normal Huh-7 cells. Cells were transfected with vectors expressing Flag-tagged NS3-4A, NS4B, NS5A, NS5B, and a mutant NS5B, D318N/D319N, that does not bind pRb [8], and cultured in the presence of MG115. Cell extracts were immunoprecipitated with anti-pRb antibody, followed by immunoblotting with anti-pRb and anti-E6AP antibodies (top panels). The bottom panels show immunoblots of the cell extracts with the same antibodies.

We immunoprecipated pRb present in 2–3 cell extracts using antibody to pRb, and demonstrated that the precipitate contained detectable E6AP (Figure 6A, lane 6). In contrast, similarly prepared precipitates from the cured 2–3c cells did not contain appreciable amounts of E6AP (Figure 6A, lane 5), nor did precipitates generated from extracts of the 2–3 replicon cells using antibodies to MDM2 or Flag (Figure 6A, lanes 4 and 8). These results suggest that HCV induces the formation of a complex involving pRb and E6AP. As we had previously shown that NS5B interacts with pRb and targets it for degradation [8], we considered it likely that NS5B was responsible for the E6AP–pRb complex observed in lysates of the replicon cells. We confirmed the interaction of NS5B with pRb by demonstrating the coimmunoprecipitation of NS5B with pRb in lysates of 2–3 cells (Figure 6B, lane 6). For reasons that remain unclear, the amount of NS5B present in the pRb precipitates was markedly reduced when the replicon cells were treated with epoxomicin prior to preparation of the extracts (Figure 6B; compare lanes 5 and 6). The overall abundance of NS5B was also reduced by epoxomicin treatment (Figure 6B; compare lanes 1 versus 2, and Figure 3A and 3B), possibly reflecting nonspecific cellular toxicity and a related reduction in viral RNA replication.

To demonstrate that the formation of the pRb–E6AP complex was dependent upon NS5B and not other HCV proteins expressed by the replicon RNA, we transfected normal Huh-7 cells with vectors expressing various nonstructural HCV proteins, as shown in Figure 4C. Extracts prepared from these transfected cells were precipitated with antibody to pRb, then immunoblotted using antibody to E6AP. Only expression of wild-type NS5B resulted in the formation of a complex between pRb and E6AP (Figure 6C, lane 5). Importantly, this was not observed in cells expressing an NS5B mutant, D318N/D319N, that fails to bind pRb [8] (Figure 6C; compare lanes 5 and 6), or in cells ectopically expressing other nonstructural proteins of the virus: NS3-4A, NS4B, and NS5A. Taken together, the data shown in Figure 6 indicate that NS5B forms a complex with pRb and E6AP. Consistent with the results of the siRNA knockdown experiments described above, these data provide strong support for a role for E6AP in the NS5B-dependent degradation of pRb.

### A Dominant-Negative E6AP Mutant Restores pRb Abundance in Replicon Cells

**Figure 7:**
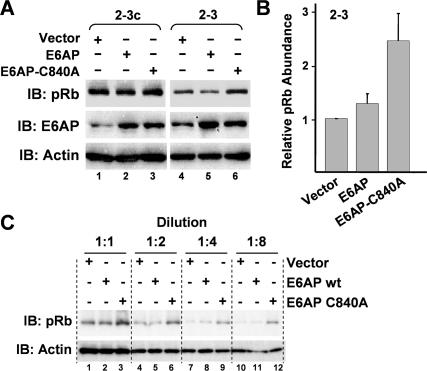
HCV-Induced Degradation of pRb Is Dependent upon the Ubiquitin Ligase Activity of E6AP (A) Immunoblot analysis showing that ectopic expression of a dominant-negative E6AP mutant (E6AP-C840A) partially restores pRb abundance in HCV replicon cells. 2–3 and cured 2–3c cells were transfected with vectors expressing wild-type E6AP and E6AP-C840A, which contains a mutation within the catalytic site of the ubiquitin ligase and acts as dominant-negative [35]. Cell extracts were immunoblotted with anti-pRb and anti-E6AP antibodies. Actin was used as a loading control. (B) Mean ± standard deviation of values for abundance of pRb in cells transfected with wild-type and mutant E6AP expression vectors obtained in three replicate experiments carried out as shown in (A), relative to abundance in cells transfected with an empty vector. (C) Quantitative pRb immunoblots of serial 2-fold dilutions of lysates of 2–3 cells transfected with expression vectors for wt E6AP, E6AP-C840A, or empty vector.

To determine whether E6AP in fact functions as an E3 ligase for pRb, we assessed the ability of recombinant E6AP to direct the ubiquitination of pRb in a reconstituted in vitro ubiquitination reaction using recombinant E1 and E2 proteins. However, these experiments failed to demonstrate ubiquitination of pRb by E6AP, either in the presence or absence of NS5B (Figure S3). These results suggest that E6AP may not be responsible for the NS5B-dependent ubiquitination of pRb, or that this process requires another cellular or viral protein partner that was not included in the reconstituted in vitro ubiquitination reaction. To distinguish between these possibilities, we transfected the 2–3 replicon cells with vectors expressing either wild-type E6AP or a dominant-negative E6AP protein, E6AP-C840A, that contains a single amino acid substitution within the C-terminal HECT domain, ablating its E3 ligase activity [35]. Quantitation of immunoblots from three independent experiments demonstrated that the overexpression of E6AP-C840A resulted in a reproducible increase in the abundance of pRb in the 2–3 replicon cells (Figure 7A [compare lanes 4 versus 6] and 7B). This increase in pRb abundance was clearly apparent in immunoblots of serial 2-fold dilutions of the cell lysates (Figure 7C). In contrast, overexpression of wild-type E6AP caused either no change or a slight increase in pRb abundance in 2–3 cells, and did not appreciably alter pRb expression in the cured 2–3c cells (Figure 7A). These results provide additional evidence that E6AP is required for NS5B-dependent ubiquitination of pRb, and are consistent with the effects of siRNA knockdown of E6AP (Figure 5) and the presence of an NS5B–E6AP–pRb complex in lysates of the replicon cells (Figure 6). We conclude that E6AP is the E3 ligase responsible for NS5B-dependent ubiquitination of pRb in vivo.

## Discussion

We have shown here that pRb is ubiquitinated and degraded in a proteasome-dependent fashion in cultured human hepatoma cells infected with HCV. These observations enhance the biological relevance of prior studies showing that pRb is downregulated by the HCV polymerase protein, NS5B, expressed by autonomously replicating RNA replicons [8]. In showing that the cellular E6AP and viral NS5B proteins form a complex that regulates pRb abundance, we have also provided an enhanced mechanistic understanding of this process.

E6AP was identified originally as a ubiquitin ligase that downregulates p53 in cells expressing the HPV E6 protein [28,36]. The E6–E6AP complex targets additional proteins for ubiquitination, including a set of PDZ domain proteins [37,38] and NFX1–91, a repressor of the hTERT promoter [39]. Interestingly, E6AP has also been shown recently to ubiquitinate and regulate the stability of the HCV core protein [32]. The partial restoration of pRb abundance we observed in the 2–3 replicon cells following either siRNA knockdown of E6AP (Figure 5) or overexpression of a dominant-negative E6AP mutant (E6AP-C840A; Figure 7) provides strong evidence that the ubiquitin ligase activity of E6AP is required for HCV regulation of pRb. Importantly, neither E6AP knockdown nor E6AP-C840A expression altered the abundance of pRb in the clonally related 2–3c cells that had been cured of the HCV replicon by prior treatment with interferon. We also found that the ability of NS5B to form a complex with E6AP is dependent upon the NS5B LH^314–318^ domain [8] that mediates the interaction of NS5B with pRb (Figure 6C; compare lanes 5 and 6). These data thus lead us to propose a model in which NS5B interacts with hypophosphorylated pRb within the cytoplasm and recruits E6AP to the complex, thereby inducing the ubiquitination and subsequent degradation of pRb via the proteasome. Such a model is consistent with the cytoplasmic redistribution of pRb, as well as the colocalization of pRb and viral nonstructural proteins (specifically NS5A) that we observed by confocal microscopy in cells infected with HCV in vitro (Figure 2A and 2B).

Several possible explanations exist for the inability of E6AP to ubiquitinate pRb in an NS5B-dependent fashion in reconstituted in vitro reactions (Figure S3). First, it may be that additional cellular or viral proteins are required for ubiquitination. Strong evidence exists for functionally important interactions between the NS5B polymerase and several cellular proteins other than pRb, including vesicle-associated membrane-associated proteins A and B (VAP-A and VAP-B), cyclophilin B (CypB), and protein kinase C–related kinase 2 (PRK2), which putatively regulates NS5B by phosphorylation, as well as hPLIC-1, mentioned above [27,40–43]. The deletion of a 21–amino acid C-terminal hydrophobic domain in the NS5B used in our assays (which is required for its solubility) [44,45], could also have affected the in vitro assays, potentially compromising the ability of the polymerase to undergo the conformational changes required for efficient interaction of the LH^314–318^ domain with pRb [8]. This domain overlaps the Gly-Asp-Asp motif within the active site of the NS5B polymerase, and is sequestered within the interior of the protein in its fully folded form [46].

Prior studies indicate that pRb abundance is regulated by several different mechanisms, reflecting in turn its role as a master regulator of the cell cycle. The E3 ligase MDM2 plays a prominent role in its regulation in the absence of HCV infection [23,24]. While we observed a variable increase in pRb abundance following siRNA knockdown of MDM2 in the 2–3 replicon cells (Figure 5A; compare lanes 6–7 and 10), this effect was typically less than that observed with E6AP knockdown (compare lanes 9 and 10). Moreover, in contrast to E6AP, we were not able to demonstrate an interaction between NS5B and MDM2 (unpublished data). pRb is also targeted for proteasome-mediated degradation by the E7 oncoprotein expressed by high-risk HPVs [47]. Although recognized for many years, the mechanism underlying its regulation by E7 has remained for the most part obscure. However, an active cullin 2 ubiquitin ligase complex has been reported recently to be associated with the HPV type 16 E7 protein, and has been implicated in this process [48]. The Epstein-Barr virus latent antigen 3C mediates degradation of pRb by recruiting the SCF^Skp2^ ubiquitin ligase [49]. Thus, the steady-state abundance of pRb appears to be regulated through the actions of three distinct E3 ligases: by MDM2 in normal cells, by SCF^Skp2^ in Epstein-Barr virus–infected B cells, and (as our data suggest) by E6AP in HCV-infected hepatocytes. The human cytomegalovirus pp71 protein also promotes the degradation of pRb and its associated family proteins, but does so in a proteasome-dependent, ubiquitin-independent manner [50]. In addition, gankyrin, an oncogenic ankyrin-repeat protein that is overexpressed in most HCCs, associates with pRb and reduces its stability [51].

While the abundance of pRb may be regulated through a diversity of mechanisms, its activity in controlling E2F transcription factors is largely regulated by phosphorylation [14]. The sequential phosphorylation of pRb plays a pivotal role in the G_1_/S-phase transition. In its hypophosphorylated state, pRb sequesters and represses the E2F family of transcription factors [13]. Mitogenic growth factors induce phosphorylation of pRb by activation of cyclin D–Cdk4/6 and cyclin E/Cdk2 complexes [52]. This results in release of E2F proteins from pRb, and promotes transcriptional activation of genes required for S-phase and DNA replication. It also appears to promote the nuclear export of pRb, at least in some cell types [21]. Conversely, growth-inhibitory signals reduce cyclin levels or induce Cdk inhibitors, resulting in decreased cyclin/Cdk activity, hypophosphorylation of pRb, and, subsequently, repression of E2F-target genes. Importantly, some ubiquitin ligases are known to catalyze ubiquitination in a phosphorylation-dependent manner. For example, ubiquitination of p27^Kip1^ is triggered by phosphorylation on Thr187 by Cdk2–cyclin E/A kinase complexes [53,54]. Phosphorylated p27^Kip1^ is then recognized by SCF^Skp2^, which polyubiquitinates p27^Kip1^, targeting it for degradation by the proteasome. At present, we have no direct evidence that phosphorylation influences the ubiquitination and degradation of pRb. However, the relatively low abundance of cytoplasmic phospho-pRb in epoxomicin-treated hepatoma cells infected with HCV (Figure 2D, frame vi) suggests that NS5B may interact preferentially with hypophosphorylated pRb. pRb is also subject to acetylation and sumoylation, both of which regulate pRb function within the cell cycle [55,56], and could also influence how pRb is ubiquitinated. The effect of such modifications on the regulation of pRb by HCV will require further study.

Since the ectopic expression of NS5B stimulates the activity of E2F-responsive promoters, S-phase entry, and cellular proliferation [8], it seems likely that HCV regulation of pRb abundance may enhance the proliferative rate of infected hepatocytes. This virus–host interaction may have evolved because it favors viral RNA replication, which is known to be stimulated in proliferating cells in vitro [57,58]. A more important question, however, is whether the NS5B-mediated degradation of pRb could contribute to the development of HCC in patients with chronic HCV infection.

While the manner in which the expression of E7 by high-risk papillomaviruses contributes to cervical cancer [59] may hold some analogies for HCV, it is likely that HCV regulation of pRb promotes the development of liver cancer in a more indirect fashion. In addition to its role in controlling the G_1_- to S-phase transition, pRb regulates mitotic checkpoints that are critical controls in prevention of cancer, as they allow cells to repair chromosomal damage before DNA replication or cell division. The risk of oxidative chromosomal DNA damage is likely to be enhanced due to inflammation within the liver during chronic hepatitis C, increasing the importance of these mitotic checkpoints. However, to be fully functional, these checkpoints require competent p53 and pRb pathways [60,61]. Expression of the E6 and E7 proteins from high-risk HPVs can override DNA damage-induced G_1_ arrest [62], while E6 proteins from high-risk HPVs are also able to compromise the G_2_/M DNA damage checkpoint [63]. We have shown previously that NS5B-mediated degradation of pRb results in an upregulation of the activity of the E2F-responsive Mad2 promoter [8]. Importantly, deregulation of Mad2, an essential component of the mitotic spindle checkpoint, leads to aneuploidy and an increased risk of tumors, including hepatomas, in mice [64,65]. Thus, while it remains to be proven that HCV infection disrupts mitotic checkpoints through downregulation of pRb, it is a reasonable hypothesis. By downregulating pRb abundance, HCV infection would both stimulate hepatocellular proliferation within a local environment rich in reactive oxygen species and also impair the ability of the cell to respond appropriately to DNA damage. The net result would be increased chromosomal instability. Such a hypothesis is consistent with the diversity of chromosomal abnormalities found in HCC, as well as the typically lengthy period spanning the onset of HCV infection to the development of HCC, and should provide a useful framework for future investigations.

## Materials and Methods

### Cells.

Human hepatoma cells Huh-7 and Huh-7.5 [66] were grown in Dulbecco modified Eagle medium (Cellgro, http://www.cellgro.com/) supplemented with 10% (v/v) heat-inactivated fetal bovine serum, 100 U/ml penicillin G, and 100 μg/ml streptomycin, at 37 °C in a humidified atmosphere with 5% (v/v) CO_2_. The NNeo/C-5B 2–3, Huh-7–derived cell line containing autonomously replicating, genome-length, dicistronic, selectable HCV RNAs derived with the genotype 1b HCV-N strain were cultured with 500 μg/ml G418, as described previously (Cellgro) [22]. Its companion, interferon-cured progeny cell line 2–3c, was generated and maintained as described previously, and contains no HCV RNA [58].

### Virus.

Cell culture–infectious genotype 1a H77S and genotype 2 JFH1 viruses were harvested from the supernatant fluids of cultures of RNA transfected Huh-7.5 cells, and stored at −80 °C until use [16,20]. For experiments with JFH1 virus, cells were inoculated at a multiplicity of infection (MOI) of 1–2, and virus was allowed to adsorb to cells for 6–12 h at 37 °C prior to replacement of media. pRb abundance and cellular localization was ascertained by immunoblotting and confocal microscopy 48–120 h after infection. H77S infections were carried out at an MOI of ∼0.01 due to the lower efficiency of virus production, and cells were examined by confocal microscopy only.

### Plasmids.

pCMV6-hE6AP, containing full-length cDNA of human E6AP cloned into the mammalian expression vector pCMV6, was purchased from OriGene. A dominant-negative mutant, E6AP C840A, was generated by PCR mutagenesis using pCMV6-hE6AP as a template with the primers 5′-GCC TTT AAT GTG CTT TTA CTT CCG G-3′ and 5′-AGT ATG AGA TGT AGG TAA CCT TTC-3′. Human ubiquitin B precursor cDNA was purchased from OriGene (http://www.origene.com/), and cDNA representing the mature ubiquitin was subcloned into the pGEM-T Easy cloning vector (Promega, http://www.promega.com/) after amplification by PCR using the primers 5′-CCG GAA TTC ATG CAG ATC TTC GTG AAA ACC CTT AC-3′ and 5′-GCT CTA GAT TAA CCA CCT CTC AGA CGC AGG ACC-3′ to generate pTM-047. After confirming the sequence of both strands of the insert, pTM-047 was digested with EcoRI and XbaI, and the 0.25-kb fragment containing the ubiquitin open reading frame was subcloned into pcDNA3.1/Zeo/IRES-3xFLAG to generate a 3xFLAG-tagged ubiquitin expression vector. pEGFP-C1, pORF9-hRB1, pCMV-tag4-NS3/4A, pCMV-tag4-NS4B, pCMV-tag4-NS5A, pCMV-tag4-NS5B wt, and pCMV-tag4-NS5B D318N/D319N were constructed as described previously [8].

### Proteasome inhibitors.

Lactacystin, MG115, and epoxomicin (all from Calbiochem, http://www.emdbiosciences.com/) were prepared as solutions in DMSO. For treatment of replicon cells, 2–3 replicon and cured 2–3c cells were seeded into 6-well plates and grown to 50% confluence. Inhibitors were added to the culture media at the indicated concentrations, and cells were incubated for 10–12 h (lactacystin or MG115) or 20 h (epoxomicin), followed by preparation of cell extracts for immunoblots. For epoxomicin treatment of JFH1 virus–infected cells, the inhibitor was added 20 h prior to lysis of cells and preparation of cell extracts.

### RNA interference.

siRNA oligonucleotide SMARTpools, each containing four siRNA oligonucleotides specific for human MDM2 (M-003279–02), E6AP/UBE3A (M-005137–00), and NEDD4 (M-007178–01), and individual E6AP/UBE3A siRNAs, were purchased from Dharmacon (http://www.dharmacon.com/). Negative control siRNAs (4611 and 4613) were from Ambion (http://www.ambion.com/). For knockdown experiments, 2–3 replicon and cured 2–3c cells were grown to 30% confluence in 6-well plates, and transiently transfected with 80 nM of siRNAs using Lipofectamine 2000 (Invitrogen, http://www.invitrogen.com/) according to the manufacturer's instructions. Protein extracts were prepared for further analysis 72–120 h after transfection.

### Transfection.

Cells were seeded into 6-well plates 24 h before transfection and grown to 50% confluence. Before transfection, the culture medium was replaced with fresh medium without antibiotics. For overexpression of E6AP or E6AP C840A, 2–3 and 2–3c cells were transiently transfected with 4 μg of pCMV6 (empty vector), pCMV6-hE6AP, or pCMV6-hE6AP-C840A, along with 0.25 μg of pEGFP-C1 (Promega), using FuGENE 6 reagents (Roche Diagnostics, http://www.roche.com/). Protein extracts were prepared for immunoblots at 48 h after transfection.

### Immunoblots.

Cells were washed three times with chilled PBS, and incubated in chilled lysis buffer (20 mM Tris-HCl [pH7.5], 150 mM NaCl, 10 mM EDTA-2Na, 1% [v/v] Nonidet P-40, 10% [v/v] glycerol, and 2 mM DTT) supplemented with 1 mM PMSF and 2 μg/ml aprotinin, or complete protease inhibitor cocktail (Roche), for 30 min at 4 °C. Cell debris was pelleted by centrifugation at 13,000*g* for 30 min at 4 °C, and supernatants were used as soluble fractions. Protein concentrations were determined by the modified Bradford assay with BSA as a standard (Bio-Rad, http://www.bio-rad.com/). SDS-PAGE and subsequent immunoblotting were done as described previously [8], using mouse monoclonal antibodies against β-actin (AC-15; Sigma, http://www.sigmaaldrich.com/), GAPDH (glyceraldehyde-3-phosphate dehydrogenase; Ambion), Flag tag (M2; Sigma), MDM2 (SMP14; Santa Cruz Biotechnology, http://www.scbt.com/), pRb (G3–245; BD Biosciences, http://www.bdbioscences.com/), and ubiquitin (P4D1; Santa Cruz Biotechnology), and rabbit polyclonal antibodies against phospho-pRb 807/811 (Cell Signaling Technology, http://www.cellsignal.com/), E6AP (sc-25509; Santa Cruz Biotechnology), NEDD4 (sc-25508; Santa Cruz Biotechnology), and NS5B (A266–1; ViroGen, http://www.virogen.com/; or provided as a generous gift by Dr. Craig E. Cameron, Pennsylvania State University, State College, Pennsylvania, United States). Membranes were probed with appropriate secondary antibodies conjugated with horseradish peroxidase, visualized by ECL reagents (Amersham Pharmacia Biosciences, http://www.amersham.com/), and exposed to x-ray films.

### Indirect fluorescence microscopy.

Huh-7 2–3 and 2–3c cells were seeded into 8-well Labtek chamber slides and grown until 50%–60% confluent, with or without the addition of 20 μM of MG115. After washing twice with PBS, the cells were fixed in methanol–acetone (1:1 [vol/vol]) for 10 min at −20 °C, air-dried for 60 min at room temperature, washed twice with PBS, and incubated with blocking buffer (1% BSA in PBS) overnight at 4 °C. pRb was visualized by staining with mouse monoclonal antibody G3–245. After washing three times with PBS, slides were further incubated with a goat anti-mouse Ig secondary antibody conjugated with FITC for 1 h at room temperature. Slides were then washed three times with PBS, counterstained with diamidino-2-phenylindole 2HCl (DAPI), mounted in Vectashield mounting medium (Vector Laboratories, http://www.vectorlabs.com/), and examined with a Zeiss AxioPlan2 fluorescence microscope (http://www.zeiss.com/).

### Confocal imaging.

Huh-7 2–3 and 2–3c cells or JFH1-infected Huh-7.5 cells were cultured in Labtek chamber slides (http://www.labtek.net/) and fixed with 4% paraformaldehyde in PBS for 30 min. Cells were permeabilized with Triton X-100 (0.2%) for 15 min and blocked with 10% normal goat serum at room temperature for 1 h. Cells were then incubated with the appropriate dilutions of primary antibodies for 1 h followed by secondary antibodies for 1 h at room temperature. HCV antigen was visualized with rabbit polyclonal antibody to NS5A (a generous gift from Dr. Craig E. Cameron) followed by Alexa 594 secondary antibody conjugate, or with human polyclonal antibody and an FITC-labeled anti-human Ig secondary antibody; pRb was visualized with visualized by staining with mouse monoclonal antibody G3–245 (BD Biosciences), and phospho-pRb by rabbit polyclonal antibody against phospho-pRb 807/811 (Cell Signaling Technology) followed by secondary antibodies: goat anti-mouse Ig conjugated to FITC or goat anti-rabbit Ig conjugated to Alexa 594. Slides were washed and counterstained with DAPI, and mounted in Vectashield mounting medium, then sealed and examined with a Zeiss LSM 510 laser scanning confocal microscope within the Infectious Disease and Toxicology Optical Imaging Core at the University of Texas Medical Branch.

### Pulse-chase analysis.

Pulse-chase labeling of endogenous pRb protein was done as described previously [8].

### Coimmunoprecipitation analysis.

For analysis of the interaction between pRb and E6AP in normal Huh-7 cells, cells were grown to 50% confluence in 10-cm dishes and transfected with 5 μg of pCMV-tag4, pCMV-tag4-NS3/4A, pCMV-tag4-NS4B, pCMV-tag4-NS5A, pCMV-tag4-NS5B wt, and pCMV-tag4-NS5B D318N/D319N. At 48 h after transfection, 20 μM of MG115 was added to the transfected cells for 10 h. Cells were lysed in 1 ml of IP lysis buffer (20 mM Tris-HCl [pH7.5], 150 mM NaCl, 10 mM EDTA-2Na, 1% [v/v] NP-40, 10% [v/v] glycerol, 1 mM PMSF, and 2 μg/ml aprotinin), and extracts were prepared as described above. IP was performed with 500 μg of extracts using anti-pRb monoclonal antibodies, as described previously, and immunoprecipitated proteins were analyzed by immunoblot [8]. For analysis of the interaction between pRb and E6AP in HCV replicon cells, 2–3 replicon and 2–3c cured cells were lysed in IP lysis buffer, and soluble extracts were prepared. IP was performed with 500 μg of extracts using 1 μg of anti-FLAG (M2; Sigma), anti-pRb (G3–245; BD Biosciences), or anti-MDM2 (SMP14; Santa Cruz Biotechnology) monoclonal antibody, and immunoprecipitated proteins were analyzed by immunoblot.

For analysis of the interaction between NS5B and ubiquitinated pRb, NNeo/C-5B 2–3 and 2–3c cured cells were transfected with 3xFLAG-tagged ubiquitin expression vector, and treated with DMSO or 20 μM MG115 for the last 10 h. At 48 h after transfection, cells were lysed in IP lysis buffer, and soluble extracts were prepared. IP was carried out using 500 μg of extracts and anti-FLAG monoclonal antibody, and immunoprecipitated proteins were analyzed by immunoblot.

### Cellular ubiquitination assays.

For detection of ubiquitinated pRb, 2–3 replicon cells and 2–3c cured cells were cultured to 50% confluence in 10-cm dishes and transfected with 5 μg of 3xFLAG-tagged ubiquitin expression vector. At 48 h after transfection, cells were treated with DMSO, 10 μM of lactacystin, or 20 μM of MG115 for 10 h, and then lysed in IP lysis buffer supplemented with 1 mM NaF, 1 mM Na_3_VO_4_, 4 mM N-ethylmaleimide, and 6 nM ubiquitin aldehyde (ubiquitination buffer), and soluble extracts were prepared. A total of 500 μg of extracts were used for IP with anti-FLAG monoclonal antibodies, and immunoblots were carried out with anti-pRb or antiubiquitin monoclonal antibodies.

For detection of NS5B-dependent ubiquitination of pRb, normal Huh-7 cells were cultured to 50% confluence in 6-well plates, and transfected with 2 μg pCMV-tag4, pCMV-tag4-NS3/4A, pCMV-tag4-NS4B, pCMV-tag4-NS5A, pCMV-tag4-NS5B wt, and pCMV-tag4-NS5B D318N/D319N. At 48 h after transfection, 20 μM of MG115 was added to the transfected cells for 10 h. Cells were lysed in ubiquitination buffer, and soluble extracts were prepared. IP was carried out using anti-pRb monoclonal antibodies, followed by immunoblotting with monoclonal antiubiquitin antibody.

### In vitro ubiquitination assays.

Reconstituted, in vitro ubiquitination reactions were carried out using purified recombinant pRb (Abcam, http://www.abcam.com/), recombinant NS5B protein with a 21–amino acid C-terminal deletion (Replizyme, http://www.replizyme.com/), and recombinant E6AP produced in baculovirus, essentially as described [35].

## Supporting Information

Figure S1 Quantitative Immunoblot for pRb in Lysates of Huh-7 Cells Prepared 120 h Following Mock Infection or Infection with JFH1 Virus Two-fold dilutions of lysates from mock-infected or JFH1-infected cells were blotted and probed for pRb, NS5B, and GAPDH (see Figure 1B). Densitometry of the pRb and GAPDH bands detected in the 1:2 and 1:4 lysate dilutions suggested a 63%–70% reduction in pRb abundance in the infected cells versus uninfected cells.
(926 KB TIF)

Figure S2 Inhibition of Proteasomes with MG115 Partially Restores pRb Abundance in NNeo-C5B/2–3 HCV Replicon Cells, but Has No Effect on pRb Abundance in Interferon-Cured, HCV-Negative 2–3c Cells 2–3 and 2–3c cells were treated with 0, 10, or 20 μM MG115 for 8 h, followed by lysis and immunoblot analysis of pRb and NS5B. GAPDH was used as a loading control.
(725 KB TIF)

Figure S3 E6AP Does Not Mediate NS5B-Dependent Ubiquitination of pRb In Vitro The reconstituted cell-free reaction included purified recombinant pRb (and purified p53 as a control), E1, E2, and E6AP proteins, with and without purified, recombinant NS5B (with a 21–amino acid C-terminal deletion to improve its solubility) or HPV E6 proteins. Results confirmed the ubiquitin ligase activity of the recombinant E6AP protein by demonstrating the production of high-molecular-mass ubiquitinated protein (“Ubi-X”) in reactions containing both the HPV E6 protein and p53 as a substrate (compare lanes 5 and 6). While a small amount of ubiquitinated pRb was generated in reaction mixes containing E1, E2, and E6AP (compare lanes 1 and 2), this was not increased by the addition of NS5B (lane 3). The data shown are representative of three independent experiments carried out under similar conditions. Similar experiments, using as substrate pRb that had been translated in vitro in rabbit reticulocyte lysates, failed to demonstrate NS5B-dependent ubiquitination of pRb (unpublished data).
(1.3 MB TIF)

### Accession Numbers

The Entrez Protein (http://www.ncbi.nlm.nih.gov/entrez/query.fcgi?db=Protein) accession numbers for the proteins discussed in this study are as follows: pRb (NP_000312), E6AP (NP_061828), MDM2 (NP_002383), NEDD4 (NP_006145), ubiquitin (NP_061828), HCV-N full-length polyprotein (AAD44719), and JFH1 full-length polyprotein (BAB32872).

## Abbreviations

E6AP: E6-associated protein
GAPDH: glyceraldehyde-3-phosphate dehydrogenase
HCC: hepatocellular carcinoma
HCV: hepatitis C virus
HPV: human papillomavirus
IP: immunoprecipitation
MDM2: murine double minute 2 protein homolog
MOI: multiplicity of infection
NEDD4: neural precursor cell–expressed developmentally downregulated protein 4
NS5B: nonstructural protein 5B
pRb: retinoblastoma susceptibility protein
