# Supplementary figures and images for: Hepatitis C Virus Induces E6AP-Dependent Degradation of the Retinoblastoma Protein
Source: PLoS Pathog. 2007 Sep 28;3(9):e139. doi: 10.1371/journal.ppat.0030139 (PMC2323300; doi:10.1371/journal.ppat.0030139)

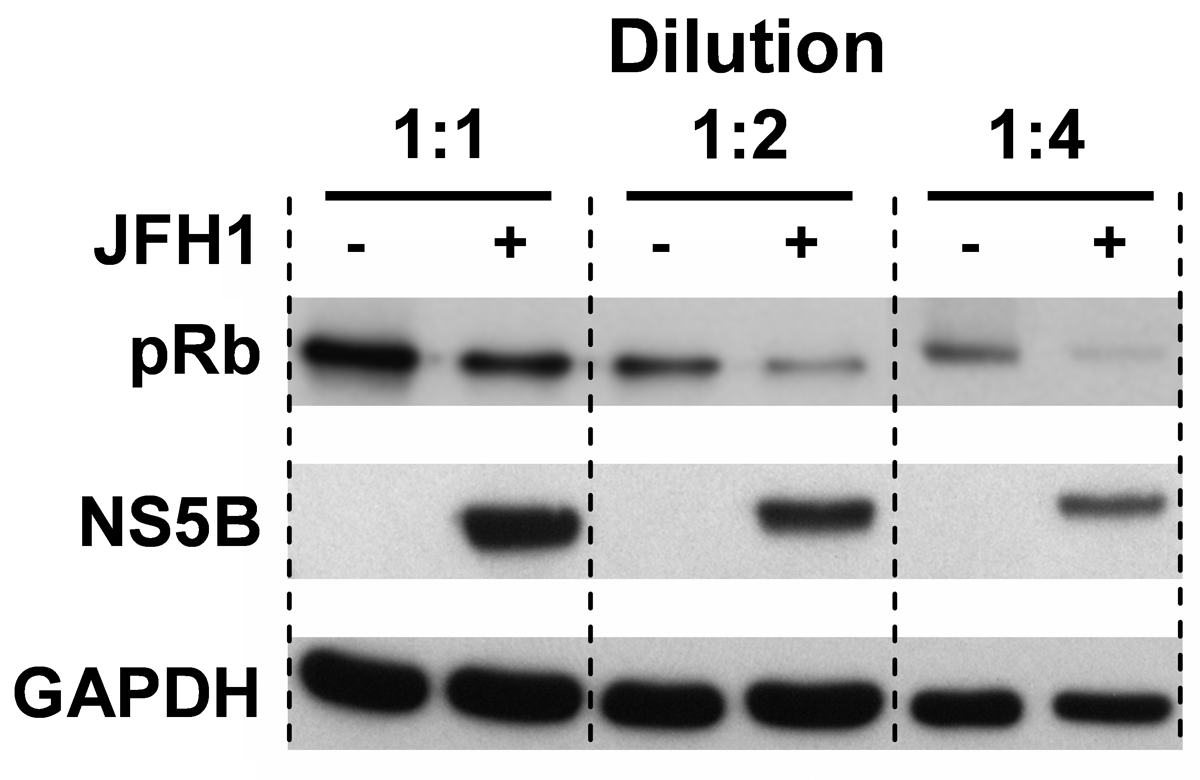

Supplement: Figure S1 — Two-fold dilutions of lysates from mock-infected or JFH1-infected cells were blotted and probed for pRb, NS5B, and GAPDH (see Figure 1B). Densitometry of the pRb and GAPDH bands detected in the 1:2 and 1:4 lysate dilutions suggested a 63%–70% reduction in pRb abundance in the infected cells versus uninfected cells. (926 KB TIF) [file ppat.0030139.sg001.tif]

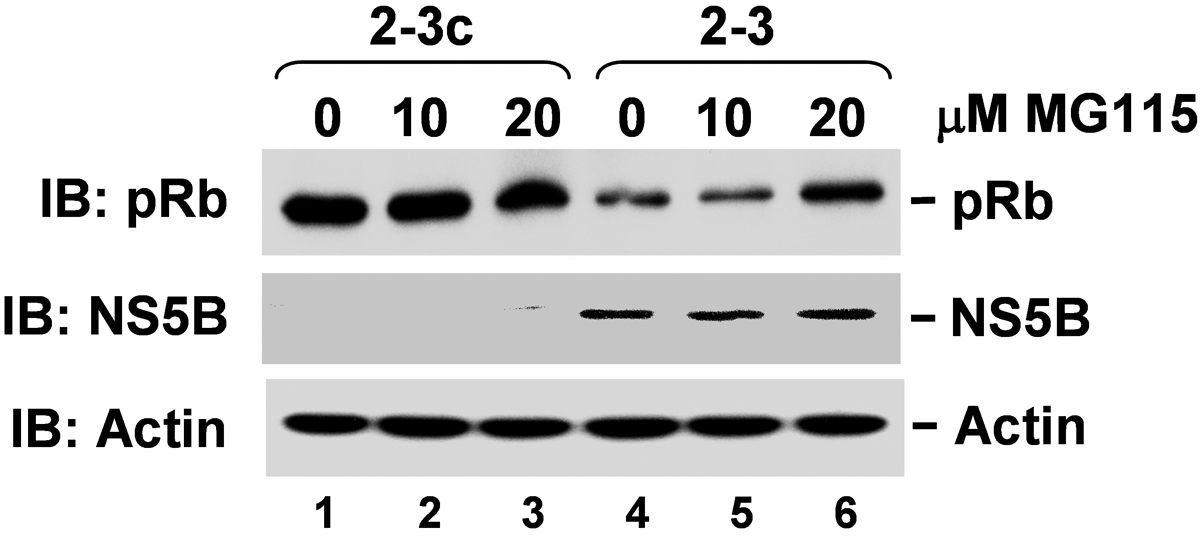

Supplement: Figure S2 — 2–3 and 2–3c cells were treated with 0, 10, or 20 μM MG115 for 8 h, followed by lysis and immunoblot analysis of pRb and NS5B. GAPDH was used as a loading control. (725 KB TIF) [file ppat.0030139.sg002.tif]

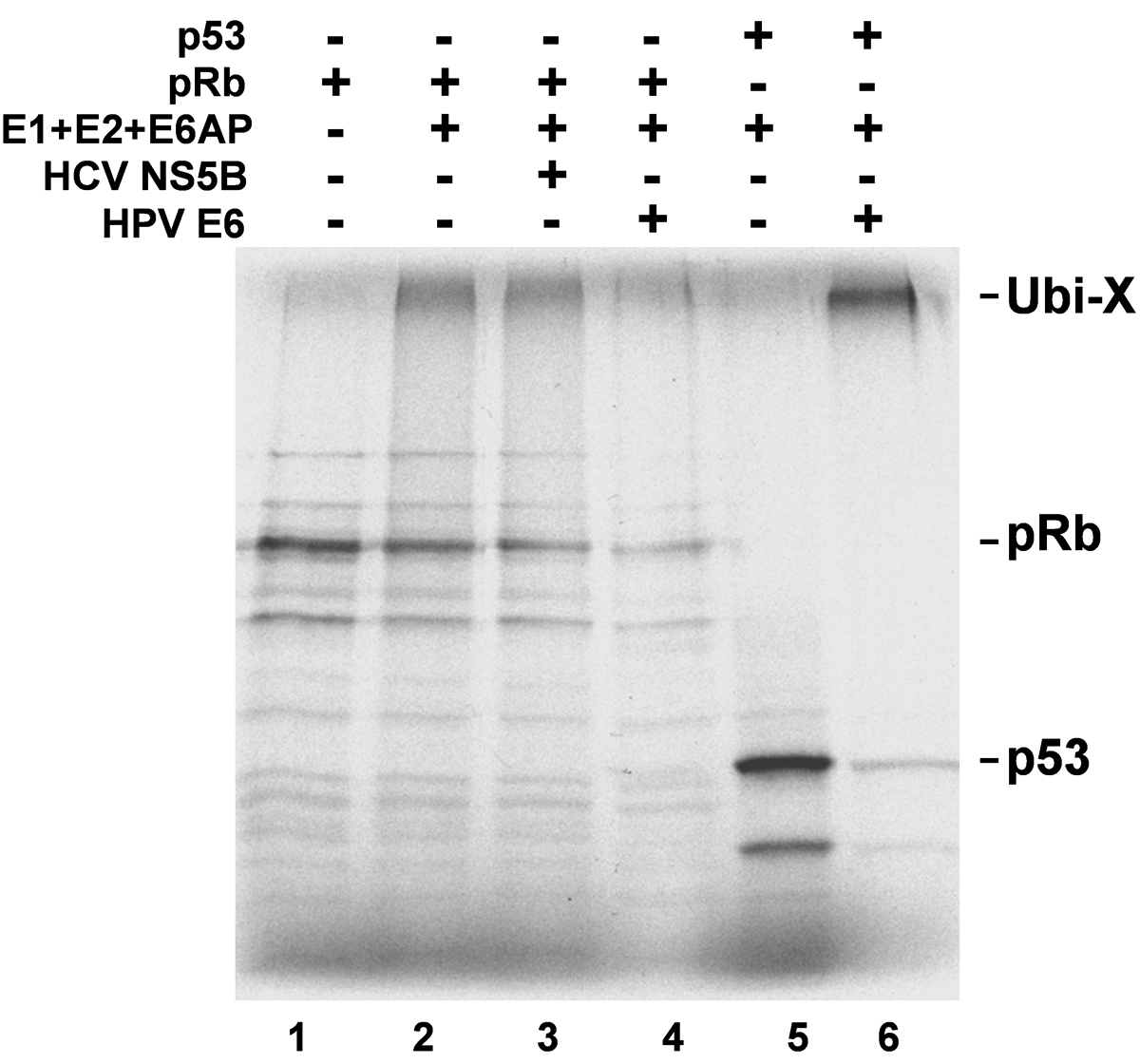

Supplement: Figure S3 — The reconstituted cell-free reaction included purified recombinant pRb (and purified p53 as a control), E1, E2, and E6AP proteins, with and without purified, recombinant NS5B (with a 21–amino acid C-terminal deletion to improve its solubility) or HPV E6 proteins. Results confirmed the ubiquitin ligase activity of the recombinant E6AP protein by demonstrating the production of high-molecular-mass ubiquitinated protein (“Ubi-X”) in reactions containing both the HPV E6 protein and p53 as a substrate (compare lanes 5 and 6). While a small amount of ubiquitinated pRb was generated in reaction mixes containing E1, E2, and E6AP (compare lanes 1 and 2), this was not increased by the addition of NS5B (lane 3). The data shown are representative of three independent experiments carried out under similar conditions. Similar experiments, using as substrate pRb that had been translated in vitro in rabbit reticulocyte lysates, failed to demonstrate NS5B-dependent ubiquitination of pRb (unpublished data). (1.3 MB TIF) [file ppat.0030139.sg003.tif]
